# Effects of a new-generation hybrid contact lens on visual performance and vision-related quality of life in patients with keratoconus

**DOI:** 10.5935/0004-2749.20230001

**Published:** 2023

**Authors:** Sait Coskun Ozcan, Deniz Ozarslan Ozcan

**Affiliations:** 1 Department of Ophthalmology, Tayfur Ata Sökmen Faculty of Medicine, Mustafa Kemal University Hatay Turkey.

**Keywords:** Contact lenses, Keratoconus, Refraction, ocular, Visual acuity, Quality of life, Surveys and questionnaires, Lentes de contato, Ceratocone, Refração ocular, Acuidade visual, Qualidade de vida, Inquéritos e questionários

## Abstract

**Purpose:**

The aim of this study was to evaluate the efficacy of a new-generation hybrid contact lens for improving visual outcomes and vision-related quality-of-life performance in patients with keratoconus who had intolerance or treatment failure of conventional correction methods such as the use of soft silicone-hydrogel or rigid gas-permeable contact lenses.

**Methods:**

Twenty-eight patients with keratoconus (42 eyes) were enrolled in this prospective cross-sectional study. Airflex (Swisslens) lenses were fitted in the patients’ eyes in accordance with the manufacturer’s instruction. Ophthalmologic examinations, including manifest refraction, best-corrected distance visual acuity, slit-lamp biomicroscopy, and National Eye Institute Visual Function Questionnaire 25 (NEI-VFQ-25) assessment, were performed at baseline and the 6-month visit.

**Results:**

An adequate fit was achieved in 39 eyes (92.9%) of 26 patients. Six eyes of 3 patients were excluded from the study owing to discontinuation of lens wearing. The mean age of the successful wearers was 20.3 ± 4.9 years. The mean best-corrected distance visual acuity was statistically significantly improved from 0.62 ± 0.30 to 0.11 ± 0.06 logMAR with the Airflex hybrid contact lenses (p<0.001). The mean overall composite NEI-VFQ-25 score statistically significantly increased with the Airflex hybrid contact lenses at the 6-month visit as compared with that at baseline (from 77.1 ± 16.3 to 90.9 ± 7.3, p=0.036). Statistically significantly better scores were obtained with the Airflex hybrid contact lenses in all the NEI-VFQ-25 subscale items (all p<0.05). No significant adverse effects were observed.

**Conclusions:**

New-generation hybrid contact lenses can be used as an effective alternative for correction of irregular astigmatism in patients with keratoconus who have intolerance or treatment failure of conventional methods. Significant improvement in vision-related quality-of-life in patients with keratoconus can be achieved with these lenses.

## Abstract (pt)

**Objetivo:**

Este estudo tem como objetivo avaliar a eficácia de lentes de contato híbridas de nova geração nos resultados visuais e na qualidade de vida relacionada à visão em pacientes com ceratocone com intolerância ou insucesso dos métodos de correção habituais, tais como lentes flexíveis de silicone-hidrogel ou rígidas permeáveis a gases.

**Métodos:**

Foram incluídos neste estudo transversal prospectivo 42 olhos de 28 pacientes com ceratocone. Uma lente Airflex (Swisslens) foi aplicada nos olhos de acordo com as instruções do fabricante. Um exame oftalmológico, incluindo refração manifesta, melhor acuidade visual corrigida para longe, biomicroscopia com lâmpada de fenda e a aplicação do *National Eye Institute Visual Function Questionnaire-25* (NEI-VFQ-25), foi realizado no início do estudo e na visita de 6 meses.

**Resultados:**

Foi possível obter um ajuste adequado em 39 olhos (92,9%) de 26 pacientes. Foram excluídos do estudo 6 olhos de 3 pacientes devido à cessação do uso de lentes. A idade média dos usuários bem-sucedidos era de 20,3 ± 4,9 anos. A média da melhor acuidade visual corrigida para longe foi melhorada estatisticamente de 0,62 ± 0,30 para 0,11 ± 0,06 logMAR com as lentes de contato híbridas Airflex (p<0,001). A pontuação média geral composta no questionário NEI-VFQ-25 aumentou de forma estatisticamente significativa com a lente de contato híbrida Airflex na visita de 6 meses, em comparação com a pontuação inicial (de 77,1 ± 16,3 para 90,9 ± 7,3, p=0,036). As lentes de contato híbridas Airflex apresentaram pontuações melhores com significância estatística em todos os itens das sub-escalas do NEI-VFQ-25 (todos com p<0,05). Nenhum efeito adverso significativo foi observado.

**Conclusões:**

Lentes de contato híbridas de nova ge ração podem ser usadas como uma alternativa eficaz para a correção do astigmatismo irregular em pacientes com ceratocone com intolerância ou insucesso dos métodos habituais. Com essas lentes, pode-se alcançar uma melhora significativa na qualidade de vida relacionada à visão em pacientes com ceratocone.

## INTRODUCTION

Keratoconus is a progressive, non-inflammatory ectasia that causes corneal protrusion, myopia, and irregular astigmatism^(1)^. The disease generally occurs in adolescence and young adulthood and is characterized by loss of vision and ocular discomfort. Various options can be applied to improve visual acuity depending on the severity of the keratoconus, such as glasses, soft and rigid contact lenses, intracorneal ring implantation, and corneal transplantation. Contact lenses, which cause temporary complications that do not threaten vision, have been reported to have been used as the main treatment method for keratoconus^(2)^.

New contact lens materials and designs are available in clinical practice for optical correction of challenging corneas in recent years^(3)^. Hybrid contact lenses (HCLs) increase visual acuity significantly with their rigid gas-permeable (RGP) center and provide greater comfort and better centralization than RGP lenses owing to their soft skirt^(4)^. Currently, the Airflex (Swisslens, Switzerland) lens is one of the latest-generation and most advanced option among these lenses.

Furthermore, keratoconus is associated with impaired vision-related quality of life (VRQOL) that progressively worsens over time^(5,6)^. As the disease progresses, daily activities such as driving, reading, and watching television can be restricted, and the daily lives and emotional well-being of the patients are seriously affected. Although the impacts of previous contact lenses on the VRQOL and satisfaction of patients with keratoconus have been investigated^(7,8)^, to the best of our knowledge, the effects of new-generation HCLs have not been studied. Thus, in this study, we aimed to evaluate the effects of the Airflex HCL on visual performance and VRQOL in patients with keratoconus.

## METHODS

### Patient data and ocular examination

This prospective cross-sectional study was conducted with patients with a diagnosis of keratoconus who were followed up in the cornea service of the Hatay Mustafa Kemal University Hospital Ophthalmology Clinic. Patients with keratoconus who were successfully fitted with the Airflex HCL and followed up for 6 months were enrolled in the study. This study was conducted in accordance with the principles of the Declaration of Helsinki. Each participant was informed about the purpose of the study and provided written informed consent. Approval from the local ethics committee was obtained.

All the patients received a detailed opthalmic evaluation, including manifest refraction, uncorrected visual acuity (UDVA) and best-corrected distance visual acuity (BDVA) with spectacles, slit-lamp biomicroscopy, and corneal topography (Sirius, CSO Inc, Florence, Italy). A diagnosis of keratoconus was made on the basis of corneal thinning and ectasia detected after clinical and topographic evaluations by a corneal specialist. The Amsler-Krumeich classification was used to grade the severity of keratoconus^(9)^.

Patients with keratoconus who had a history of intolerance or treatment failure with soft silicone-hydrogel or RGP contact lenses were included in the study. Patients with systemic diseases such as diabetes mellitus and autoimmune diseases; coexisting ocular disorders, including dry eyes; and histories of ocular trauma and chronic medical treatment that could affect the ocular surface and VRQOL outcomes were excluded from the study.

### Fitting procedures

**Figure 1:**
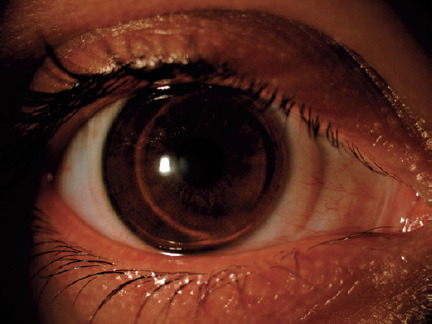
Slit-lamp biomicroscopy photograph of a patient with a keratoconic eye wearing an Airflex hybrid contact lens.

The Airflex lens has a hybrid design consisting of a RGP central zone (material: Roflufocon D, Dk: 100 × 10^−11^) and a silicone-hydrogel soft skirt (material: Filcon V_3_, Dk: 50 × 10^−11^). A trial Airflex lens set was used for the fitting process. The trial set included 14 HCLs with base curves of 5.60 to 8.20 mm in 0.20 steps, a power of 0.00 to −11.00 D in 1.0 D steps, standard 0.00 skirt curves, and a standard 14.9-mm diameter. The fitting procedure was performed by a single experienced ophthalmologist in accordance with the manufacturer’s instructions. Mean keratometric values were used to determine the initial contact lens base curve. An ideal fit was considered to cover the entire corneal surface and move like a soft contact lens (approximately 0.3 mm per blink). A thin fluorescein layer in the central zone and 1- to 2-mm thin flourescein arc in the junction were observed on slit-lamp biomicroscopy during the ideal fit. A slit-lamp biomicroscopic photograph of a patient wearing an Airflex HCL is shown in figure 1. After the optimal lens determination, overrefraction was performed in the patient. The BDVA with contact lens was recorded. The trial contact lens was removed, and the eye was evaluated to ensure no corneal staining, which was the deciding factor for ordering the contact lens from the laboratory.

### National eye institute vision function questionnaire

All the patients were asked to answer the Turkish-validated version of the 25-item National Eye Institute Visual Function Questionnaire (NEI-VFQ-25) at their first visit and sixth-month follow-up examination^(10)^. The questionnarie is used to evaluate the VRQOL of patients on the basis of their symptoms. It consists of 12 dependent subscales on general health, general vision, ocular pain, near vision, distance vision, vision-specific social functioning, vision-specific menthal health, vision-specific role difficulties, vision-specific dependency, driving, color vision, and peripheral vision. In the present study, the question on driving was excluded because most participants were not driving.

### Statistical analyses

All statistical analyses were performed using SPSS version 21.0. Categorical variables were expressed as number and percentage, and quantitative variables were described using mean ± standard deviation and range. The normal distribution of the results was checked using the Shapiro-Wilk test. The Wilcoxon signed-rank test was performed to compare the outcomes before and after Airflex HCL wearing. A p value <0.05 was considered statistically significant.

## RESULTS

A total of 42 eyes of 28 patients with keratoconus were included in the study at baseline. An adequate fit could be achieved in 39 eyes (92.9%) of 26 patients, and contact lenses were ordered. During the 6-month follow-up period, 3 patients (6 eyes, 15%) were excluded from the study because they discontinued lens wearing because of handling difficulty (4 eyes) and discomfort (2 eyes). As a result, the Airflex HCL was successfully worn in 33 (78.5%) of the initial 42 eyes in this study. Table 1 demonstrates the clinical characteristics of the successful wearers. The mean age of these patients was 20.3 ± 4.9 years, and 42% of the patients were female. The mean UDVA and BDVA with spectacles were 0.93 ± 0.37 and 0.62 ± 0.30 logMAR, respectively. The mean keratometry (Kmean) value of the eyes was 47.8 ± 2.8 D (range, 43.1–53.2 D). Of the 33 keratoconic eyes, 40% were grade 1; 42%, grade 2; and 18%, grade 3 keratoconus.

**Table 1:** Demographics and clinical characteristics of the eyes included in the study (n=33; mean ± SD)

Age (years)	20.3 ± 4.9
Sex (%, female/male)	42/58
Spherical refraction (D)	-4.19 ± 4.1
Cylindrical refraction (D)	-5.24 ± 3.4
UDVA (logMAR)	0.93 ± 0.37
BDVA with spectacles (logMAR)	0.62 ± 0.30
Kmean (D)	47.8 ± 2.8
Pachymeter (µm)	433.2 ± 37.1

UDVA= uncorrected distance visual acuity; BDVA= best-corrected distance visual acuity; logMAR= logarithm of the minimum angle of resolution.

The parameters of the fitted Airflex HCLs are summarized in table 2. The mean base curve of the lenses was 6.99 ± 0.5 mm (range, 6.00-7.80 mm). Airflex HCLs with a standard diamater (14.9) and skirt curve (0.0) were used for all the eyes. The mean power of the lenses was -4.84 ± 3.1 D (range, -11.50 to 0.00 D). The mean number of trials was 1.21 ± 0.4, with 1 (61%) to 2 (39%) trial lenses.

**Table 2:** Parameters of the fitted hybrid contact lenses (n=33; mean ± SD)

Base curve (mm)	6.99 ± 0.5 (6.00-7.80)
Power (D)	-4.84 ± 3.1 (-11.50-0.00)
Diameter (mm)	14.9
Skirt curve	0.0
Number of trials	1.21 ± 0.4 (1-2)

Table 3 shows the comparison of the visual acuity before and after Airflex HCL wearing. Overall, 88% of the eyes had a BDVA >0.3 logMAR with spectacles. The mean BDVA increased statistically significantly from 0.62 ± 0.30 logMAR to 0.11 ± 0.06 logMAR with Airflex HCLs at the 6-month follow-up visit (p<0.001). In all the eyes, 0.3 logMAR or better BDVA values were obtained with the Airflex HCLs.

**Table 3:** Difference in best-corrected distance visual acuity (BDVA) before and after Airflex hybrid contact lens (HCL) wearing

	BDVA with spectacles	BDVA with a HCL	p
Visual acuity (logMAR), mean ± SD	0.62±0.30	0.11±0.06	<0.001
≤0.3 logMAR, n (%)	5 (12)	42 (100)	
0.3-1.0 logMAR, n (%)	24 (57)	0	
≥1.0 logMAR, n (%)	13 (31)	0	

logMAR= logarithm of the minimum angle of resolution.

**Figure 2:**
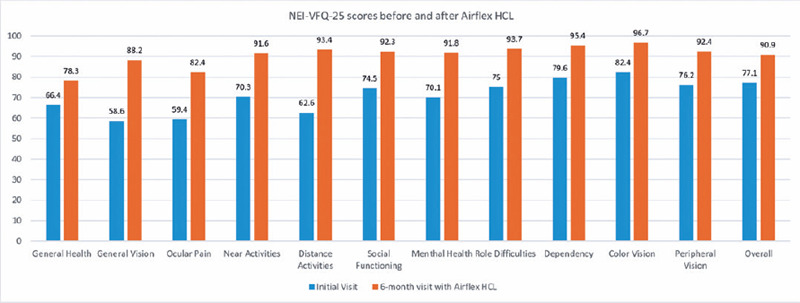
The mean National Eye Institute Visual Function Questionnaire-25 (NEI-VFQ-25) scores before and after Airflex hybrid contact lens (HCL) wearing.

Differences in the NEI-VFQ-25 scores of the patients before and after Airflex HCL wearing are shown in figure 2. The mean overall composite score in the NEI-VFQ-25 was statistically significantly improved from 77.1 ± 16.3 to 90.9 ± 7.3 with Airflex HCLs at the 6-month follow-up visit as compared with the initial visit (p=0.036). In all the NEI-VFQ-25 subscale items, a statistically significant increase in score was found with Airflex HCLs at the 6-month follow-up visit as as compared with the initial visit (Table 4). No severe adverse effects were observed during the follow-up period.

**Table 4:** Difference in NEI-VFQ-25 scores before and after Airflex HCL wearing

	Before HCL	After HCL	p
General health	66.4 ± 24.2	78.3 ± 13.8	0.041
General vision	58.6 ± 25.1	88.2 ± 10.5	<0.001
Ocular pain	59.4 ± 23.8	82.4 ± 12.3	0.006
Near activities	70.3 ± 23.1	91.6 ± 9.2	0.016
Distance activities	62.6 ± 23.5	93.4 ± 9.8	<0.001
Vision-specific social functioning	74.5 ± 20.3	92.3 ± 8.6	0.021
Vision-specific mental health	70.1 ± 19.6	91.8 ± 7.9	0.013
Vision-specific role difficulties	75.0 ± 18.4	93.7 ± 8.2	0.019
Vision-specific dependency	79.6 ± 22.5	95.4 ± 7.8	0.032
Color vision	82.4 ± 24.7	96.7 ± 6.3	0.003
Peripheral vision	76.2 ± 18.3	92.4 ± 7.7	0.029
Overall score	77.1 ± 16.3	90.9 ± 7.3	0.036

NEI-VFQ-25= National Eye Institute Visual Function Questionnaire 25; HCL= hybrid contact lens.

## DISCUSSION

Several types of contact lenses are used to correct irregular astigmatism in keratoconic eyes^(11,12)^. RGP lenses have the highest level of contribution to visual rehabilitation in keratoconus, and use of the lenses has been accepted as a gold standard treatment method for keratoconus^(13,14,15)^. However, not all patients can tolerate these lenses because of decentralization, dislocation, and discomfort problems and adverse effects such as corneal scarring and hypersensitivity. Soft contact lenses usually ensure only poor visual improvement, as they are insufficient to correct severe irregular astigmatism, as compared with RGPs^(16)^. Scleral contact lenses are large-diameter RGP lenses that do not come into contact with the cornea and neutralize irregular astigmatism through tear accumulation between the lens and corneal surface. Although scleral contact lenses provide good visual acuity, they cost higher than other lenses and can cause handling problems and discomfort during prolonged wear^(17)^.

The major advantages of HCLs are their high visual performance and ocular comfort due to the combined design of the RGP center and soft peripheral skirt. Compared with older HCL generations, the superiority of the new-generation Airflex lens is that its fitting and assessment procedures are similar to those of soft contact lenses and it does not vault over the cornea^(18)^. In this study, the mean BDVA improved significantly at the 6-month visit in the patients with keratoconus who wore Airflex HCLs. In the initial visit, the BDVA with spectacles was ≤0.3 logMAR in all the patients’ eyes. At the 6-month follow-up examination, statistically significant increases in the overall composite and all subscale sores of the NEI-VFQ-25 were observed as compared with the initial visit in the patients who wore Airflex HCLs.

Few studies have investigated the clinical performance of these particular lenses in patients with keratoconus^(18,19,20)^. Dikmetas et al. reported a significant improvement in visual function without any corneal adverse effects in keratoconus eyes with HCLs at 6-month follow-up, as in this study^(19)^. The authors suggested that HCLs may be useful as a nonsurgical treatment option in the management of advanced keratoconus. By contrast, most eyes in the present study had mild to moderate grades of keratoconus. Similar to our results, in another study that evaluate the efficacy of HCLs, the mean number of lens trials was 1.4 (1.2 in this study), the success rate was 72.5% (78% in this study), and high patient satisfaction was achieved^(18)^.

The findings from the present study strongly supports the previous research studies that demonstrated significant improvements in BDVA with new-generation HCLs in patients with keratoconus. Furthermore, this study also demonstrates the effects of these lenses on VRQOL in addition to visual acuity.

Keratoconus is a chronic disease characterized by irregular astigmatism that causes visual impairment. Decreased visual acuity has significant lifelong effects on socialization, career, and psychological health in young adult patients. In previous studies, VRQOL deficits in patients with keratoconus were demonstrated^(5,6,21)^. The NEI-VFQ is a validated instrument designed to evaluate patients’ physical and psychological well-being associated with their visual functions. Visual acuity and the corneal curvature are the main factors that affect NEI-VFQ scores^(5,21)^. In the present study, we found statistically significantly increased NEI-VFQ-25 scores at the 6-month follow-up visit in the patients with keratoconus who wore Airflex HCLs, indicating better VRQOL. The differences in general vision and distance activity subscale scores were more prominent. High visual gains resulting from the correction of myopia and irregular astigmatism with the Airflex HCL explain the significant improvement in the NEI-VFQ-25 scores. The patients with keratoconus who wore RGP lenses had better overall NEI-VFQ scores but lower ocular pain scores, which indicates more ocular discomfort than that in the non-contact lens wearers^(5,21)^. Higher subjective comfort and VRQOL scores were found in the patients with keratoconus who wore clear-cone HCLs than in the RGP wearers^(8,22)^. In this study, the combination of the soft peripheral skirt design of the Airflex HCL and high visual improvement may result in decreased ocular pain and discomfort.

The main strengths of the study were its prospective design and the inclusion of only one type of contact lens. The relatively small sample size, shorter follow-up period, and lack of different skirt options in the fitted lenses were the limitations of this study.

In conclusion, this study demonstrates that the Airflex HCL statistically significantly increases visual acuity and VRQOL in patients with keratoconus. Although the fitting procedure of the contact lenses in patients with keratoconus is a challenging and time-consuming process, it should be performed patiently and carefully because of its significant contributions to patient quality of life. From our findings, Airflex HCL may be used as an alternative method to correct irregular astigmatism in patients with keratoconus. These results should be confirmed by further studies investigating HCLs with larger sample sizes and longer follow-up periods.
